# Multimodal Treatment and Diagnostic Modalities in the Setting of Heyde’s Syndrome: A Systematic Review

**DOI:** 10.7759/cureus.28080

**Published:** 2022-08-16

**Authors:** Dawood Jamil, Hadrian Hoang-Vu Tran, Mafaz Mansoor, Samia Rauf Bbutt, Travis Satnarine, Pranuthi Ratna, Aditi Sarker, Adarsh Srinivas Ramesh, Carlos Munoz Tello, Lubna Mohammed

**Affiliations:** 1 Internal Medicine, California Institute of Behavioral Neurosciences and Psychology, Fairfield, USA; 2 General Practice, California Institute of Behavioral Neurosciences and Psychology, Fairfield, USA; 3 Pediatrics, California Institute of Behavioral Neurosciences and Psychology, Fairfield, USA; 4 General Medicine, California Institute of Behavioral Neurosciences and Psychology, Fairfield, USA

**Keywords:** acquired von willebrand disease, gastrointestinal bleeding, transcatheter aortic valve replacement, aortic stenosis (as), intestinal angiodysplasia, heyde syndrome

## Abstract

Heyde’s syndrome encompasses the triad of aortic stenosis (AS), angiodysplasia, and acquired Von Willebrand's disease (aVWD). The disease itself is a rare association that affects a small subset of patients who suffer from aortic stenosis. Nonetheless, it represents a vital area of clinical interest and is woefully underreported in the literature. Patients with Heyde’s syndrome develop gastrointestinal bleeding (GI) as a result of angiodysplasia and due to lack of adequate hemostasis, they tend to be positively predisposed toward developing gastrointestinal hemorrhage. Due to the glaring lack of comprehensive literature on Heyde's syndrome, this systematic review aims to bridge the gap by elucidating the various diagnostic and treatment options available to clinicians for Heyde’s syndrome patients as well as to give a detailed account of the pathophysiology of the disease.

This systematic review was done in accordance with the Preferred Reporting Items for Systematic Reviews and Meta-Analysis (PRISMA) guidelines. Google Scholar, Gulf Medical University (GMU) e-library, and PubMed were thoroughly searched for studies done in the last 10 years, which corresponds with our outlined inclusion and exclusion criteria. Relevant studies were then selected on the basis of their abstracts and titles. These studies then underwent a comprehensive quality assessment in which any papers which did not meet this study’s eligibility criteria were omitted. Overall, 18 studies fulfilled the criteria of this systematic review.

## Introduction and background

Heyde's syndrome has been defined as the characteristic association between aortic stenosis (AS), gastrointestinal bleeding stemming from angiodysplasia, and acquired Von Willebrand's disease (aVWD) [1,2]. The relationship between aortic stenosis and gastrointestinal (GI) bleeding was first reported by Dr. Edward Heyde in 1958 [1]. Dr. Heyde wrote a letter to the New England Journal of Medicine, wherein he reported 10 cases of large-scale gastrointestinal bleeding in patients with concomitant calcific aortic valve stenosis [3].

Heyde's syndrome is characterized by bleeding from the GI tract and although aortic stenosis-associated bleeding is relatively common, occurring in roughly 20% of patients, the bleeding is most often seen in the mucous membranes, skin surfaces, and nasopharyngeal area. The incidence of GI in patients with aortic stenosis is much lower, between 1% and 3% [4]. The mainstay of treatment for severe symptomatic aortic stenosis is aortic valve replacement which has led to a substantial decline in Heyde's syndrome-associated bleeding. The advent of transcatheter aortic valve replacement (TAVR) in the last decade has become the standard of care for inoperable patients or patients with a high operative risk. It has led to substantial drops in the rates of gastrointestinal bleeding in these patients by causing a return to the patients' prestenotic hemodynamic state [5]. A critical development in understanding the pathophysiology of Heyde's syndrome occurred in 1992 when it was found that patients with aortic stenosis can have accompanying acquired Von Willebrand's disease [4]. This is because the aortic stenosis exerts high shear stress on large Von Willebrand factor (VWF) multimers leading to their breakdown [2]. Von Willebrand factor plays numerous roles in clot formation, especially during the early stages of hemostasis, wherein it binds to subendothelial collagen. After it is formed in megakaryocytes and endothelial cells, the Von Willebrand factor coalesces into large multimers, which are subsequently cleaved into smaller pieces that circulate in the bloodstream [2]. This is of crucial importance, as the ability of these multimers to achieve adequate hemostasis and thrombosis is directly related to their size [6]. It has been hypothesized that hemostatic alteration as a result of shear stress compounded with the fragility of GI mucosa makes patients more susceptible to GI bleeding [3].

Heyde syndrome is a complex multisystem pathology in which three systems, the cardiac, gastrointestinal, and hematologic, are all involved. According to a nationwide inpatient study between 2007 and 2014, Heyde's syndrome was found in 3.1% of patients with aortic stenosis [6]. Furthermore, throughout the seven-year study period, Heyde's syndrome patients were shown to have increased inpatient mortality and increased duration of hospitalization [6]. Therefore, it represents a vital area of clinical interest. This systematic review aimed to further explore Heyde syndrome, its pathophysiology, treatments, and its diagnostic modalities to give a cohesive overview that sheds more light on this tremendously important disease.

## Review

Methods

Methodology and Search Strategy

This systematic review was carried out in accordance with the Preferred Reporting Items for Systematic Reviews and Meta-Analysis (PRISMA) standards [7]. PubMed, Google Scholar, and Gulf Medical University e-library were the databases that were used. They were comprehensively studied using relevant regular keywords, including angiodysplasia, gastrointestinal bleeding, aortic valve stenosis, transcatheter aortic valve replacement, acquired Von Willebrand's disease, and Heyde's syndrome. Medical subject headings (MeSH) were used when exploring PubMed.

The following is the search strategy: ("angiodysplasia/blood" {majr} or "angiodysplasia/complications" {majr} or "angiodysplasia/diagnosis" {majr} or "angiodysplasia/diagnostic imaging" {majr} or "angiodysplasia/epidemiology" {majr} or "angiodysplasia/surgery" {majr} or "angiodysplasia/therapy" {majr}) and ("gastrointestinal hemorrhage/complications" {MeSH} or "gastrointestinal hemorrhage/diagnosis" {MeSH} or "gastrointestinal hemorrhage/epidemiology" {MeSH} or "gastrointestinal hemorrhage/etiology" {MeSH} or "gastrointestinal hemorrhage/prevention and control" {MeSH} or "gastrointestinal hemorrhage/therapy" {MeSH}) and ("aortic valve stenosis/complications" {MeSH} or "aortic valve stenosis/diagnosis" {MeSH} or "aortic valve stenosis/diagnostic imaging" {MeSH} or "aortic valve stenosis/epidemiology" {MeSH} or "aortic valve stenosis/physiopathology" {MeSH} or "aortic valve stenosis/surgery" {MeSH}).

The results yielded were then reviewed for their applicability; this was done by examining the titles and abstracts of the papers, irrelevant papers were then removed, and pertinent articles were further analyzed.

Inclusion and Exclusion Criteria

This paper included systematic reviews, traditional reviews, randomized control trials, cross-sectional studies, case-control studies, cohort studies, research journals, and case reports, within the last decade. Studies in languages other than English, studies before 2012, animal studies, and pediatric studies were excluded. All the studies included in the systematic review were thoroughly evaluated by two researchers according to the inclusion and exclusion criteria outlined above.

Quality Assessment

In order to evaluate the quality of the papers included, the following tools were used: for traditional reviews, the Scale for the Assessment of Narrative Review Articles (SANRA) checklist was implemented, with studies only being included if they attained a score of nine or higher. For observational and non-randomized control trials, the Newcastle-Ottawa scale was used, with studies only being included if they attained a score of 10 or higher. For case reports, the Joanna Briggs Institute's (JBI's) critical appraisal tool was used, with studies only being included if they attained a score of seven or higher. After completing the quality appraisal, 18 articles were included in this systematic review.

Results

**Figure 1:**
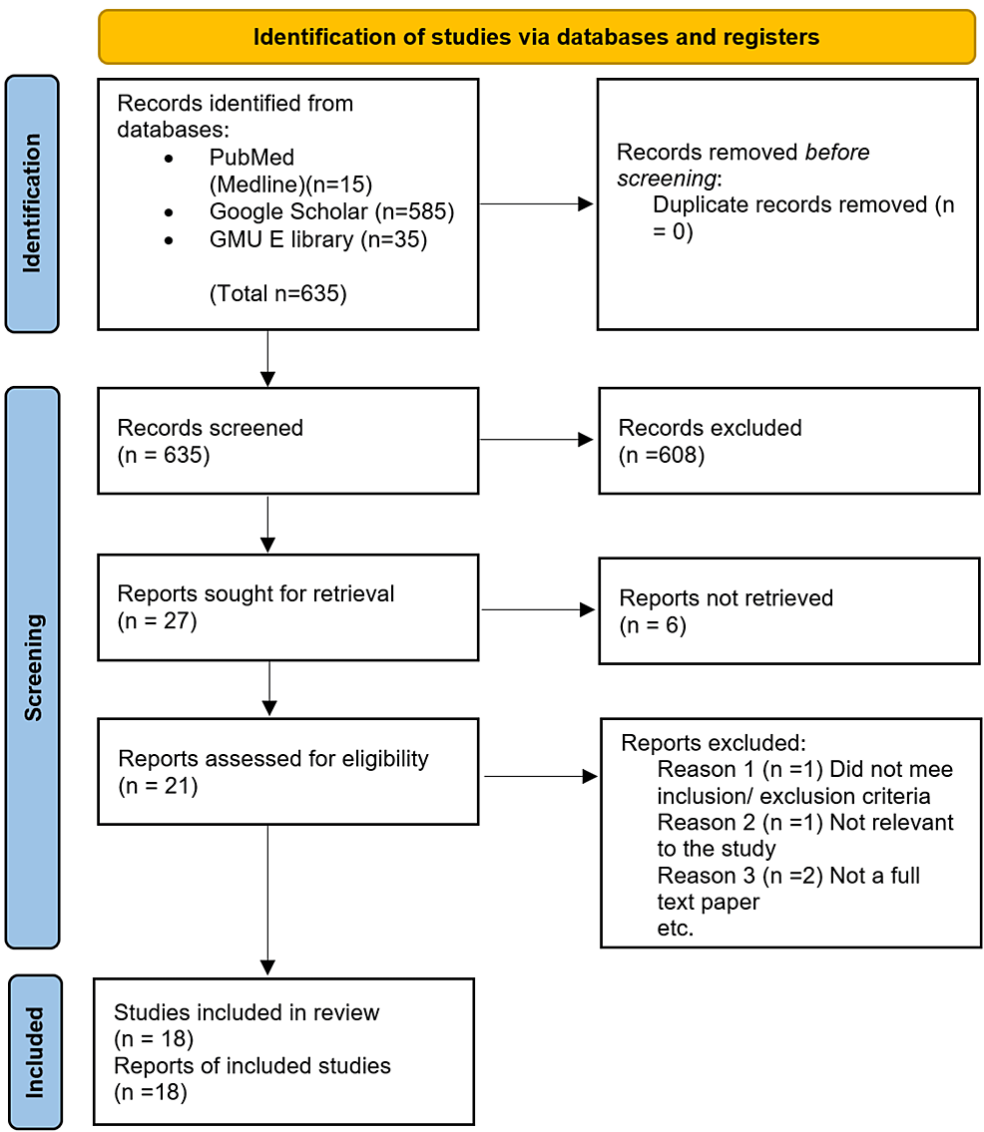
The PRISMA chart shown above illustrates the screening process used to ultimately select the studies which were included in this systematic review.

This systematic review was carried out in accordance with the PRISMA criteria, which were used to screen and narrow down studies incorporated in this systematic review, it is outlined below in Figure 1 [7].

Three databases were used to search for articles relevant to this study, PubMed, Google Scholar, and GMU e-library. A thorough search was performed using the MeSH strategy outlined above as well as relevant regular keywords which yielded a total of 634 results. From these, 21 studies were sought for retrieval; we subsequently applied our inclusion and exclusion criteria in addition to performing a quality appraisal for each study. This resulted in three studies being eliminated, and ultimately 18 studies were included in this systematic review. Table 1 shown below is a summary of the pertinent findings of the 18 studies that were included in this systematic review [1-20].

**Table 1:** Summary of the 18 studies included in this systematic review. TAVR: transaortic valvular replacement; SAVR: surgical aortic valvular replacement; GI: gastrointestinal; VWD: von Willebrand's disease; VWF: Von Willebrand Factor; TAVI: transcatheter aortic valve implantation

Primary author	Title of paper	Year of study	Number of subjects	Findings/conclusion
Lourdusamy et al. [1]	Aortic stenosis and Heyde's syndrome: a comprehensive review	2021	Not applicable	This review outlined Heyde's syndrome and compared transcatheter aortic valve replacement (TAVR) and surgical aortic valve replacement (SAVR), with TAVR having fewer perioperative complications. Furthermore, the authors shed light on the fact that concrete guidelines do not exist regarding Heyde's syndrome management and hence put forth a proposed algorithm for patient management.
Hudzik et al. [2]	Heyde syndrome: gastrointestinal bleeding and aortic stenosis	2016	One	This case was about an 82-year-old patient who presented with gastrointestinal bleeding. Upon further workup, he was found to have aortic stenosis and low Von Willebrand factor levels. He underwent transaortic valvular replacement and, at the 12-month follow-up, was found to have no further incidences of gastrointestinal bleeding.
Thompson et al. [3]	Risk of recurrent gastrointestinal bleeding after aortic valve replacement in patients with Heyde syndrome	2012	Not applicable	This paper aimed to assess the efficacy of aortic valvular replacement in decreasing the incidence of GI bleeding in Heyde's syndrome patients. The result was that aortic valvular replacement reduced the incidence of bleeding and 80% of patients had no recurrence.
Natorska et al. [4]	Increased bleeding risk in patients with valvular aortic stenosis: from new mechanisms to new therapies	2016	Not applicable	This review established a relationship between Heyde's syndrome and acquired VWD. It also summarized mechanisms of bleeding in the setting of severe aortic stenosis; furthermore, it explored treatment options and provided insight into Heyde's syndrome
Sedaghat et al. [5]	Transcatheter aortic valve implantation (TAVI) leads to a restoration of Von Willebrand factor (VWF) abnormalities in patients with severe aortic stenosis -incidence and relevance of clinical and subclinical VWF dysfunction in patients undergoing transfemoral TAVI	2017	74	The aim of this prospective cohort was to ascertain the link between Von Willebrand factor levels and patients undergoing TAVI. Seventy-four patients with severe aortic valve stenosis had their pre-TAVI VWF levels analyzed, and the result was that TAVI restored VWF levels.
Mondal et al. [6]	Heyde syndrome-pathophysiology and perioperative implications	2021	Not applicable	This paper reviewed perioperative considerations that should be taken into account prior to and during valvular replacement in Heyde's syndrome patients. This included coagulation profiles and lab tests. Furthermore, it also outlined various management options for Heyde's syndrome.
Magro [9]	Unclear anemia in an elderly subject with aortic stenosis	2015	one	This case is on a patient with aortic stenosis and angiodysplasia with concomitant iron deficiency anemia. Video capsule and endoscopic evaluation pointed towards a diagnosis of Heyde's syndrome. The patient was then successfully treated with mesenteric embolization
Johnson et al. [10]	Aortic valve stenosis, a precipitating factor of recurrent bleed in colonic angiodysplasia: a literature review	2021	Not applicable	This review article aimed to give an updated overview of Heyde's syndrome; it explained its pathophysiology and clinical presentation in addition to its diagnostic and treatment modalities.
Sami et al. [11]	Review article: gastrointestinal angiodysplasia -pathogenesis, diagnosis, and management	2013	Not applicable	This review article summarized angiodysplasia's current literature, including evolving diagnostic and treatment modalities. The authors concluded that treatment choices should be decided on a case-by-case basis.
Mohee et al. [12]	Aortic stenosis and anemia with an update on approaches to managing angiodysplasia in 2018	2020	Not applicable	This review article found that a multidisciplinary approach is optimal to ensure patient safety when managing Heyde's syndrome patients. Furthermore, it advocated for additional studies to be done on this condition.
Van Belle et al. [13]	Von Willebrand factor and management of heart valve disease	2019	Not applicable	This review explored the potential use of VWF as a biomarker for valvular heart disease in addition to exploring its use in providing real-time feedback to detect paravalvular regurgitation during the TAVR procedure. This review concluded that larger-scale studies would be required to fill existing knowledge gaps prior to widescale implementation.
De Larochellière et al. [14]	Blood disorders in patients undergoing transcatheter aortic valve replacement	2019	Not applicable	This review article highlights various hematological conditions experienced in pre- and post-TAVR patients, such as thrombocytopenia and anemia. The authors advocate that this should be taken into account as these conditions lead to a higher risk of patient mortality.
Carità et al. [15]	Aortic stenosis: insights on pathogenesis and clinical implications	2016	Not applicable	This review article describes the pathogenesis of aortic stenosis. It highlights that its progression is driven by numerous factors such as mechanical, autoimmune, and genetic. An enhanced understanding of its pathogenesis can possibly lead to the development of newer and more effective therapies for aortic stenosis
Grimard et al. [16]	Aortic stenosis: diagnosis and treatment	2016	Not applicable	Aortic stenosis is a condition commonly found in elderly patients. Initially, it is compensated; however, over time, patients become symptomatic. For patients with severe aortic stenosis, it is recommended that they undergo valvular replacement. However, for most asymptomatic patients, watchful waiting is recommended.
Zeng et al. [17]	Pathophysiology of valvular heart disease (review)	2016	Not applicable	This review drew attention to the fact that increased age leads to a rising incidence of valvular heart disease. Furthermore, it gave a detailed overview of the biological, molecular, and genetic mechanisms of aortic valvular disease pathogenesis. Inflammation plays a crucial role in eliciting calcification; this process is carried out via inflammatory mediators.
Musilanga et al. [18]	Reappraising the spectrum of bleeding gastrointestinal angioectasia in a degenerative calcific aortic valve stenosis: Heyde's syndrome	2021	Not applicable	This review explained the pathophysiology, diagnostic modalities, and treatment options for Heyde's syndrome with emphasis on pharmacological treatments such as octreotide, thalidomide, and estrogen and progesterone therapy.
Garcia et al. [19]	Heyde syndrome treated by conventional aortic valve replacement	2019	One	This case is about a 64-year-old male who presented with acute heart failure and gastrointestinal hemorrhage. Echocardiography was done, showing severe aortic stenosis, and the diagnosis of Heyde's syndrome was made. He was treated for anemia and heart failure and then underwent valve replacement surgery, after which there were no further gastrointestinal bleeding episodes.
Waldschmidt et al. [20]	Heyde syndrome: prevalence and outcomes in patients undergoing transcatheter aortic valve implantation	2021	2548	This study showed that a substantial number of patients diagnosed with Heyde's syndrome had recurrent bleeding post-valvular replacement. The author hypothesized that it might be due to residual paravalvular leakage exerting shear stress.

Discussion

Pathophysiology

**Figure 2:**
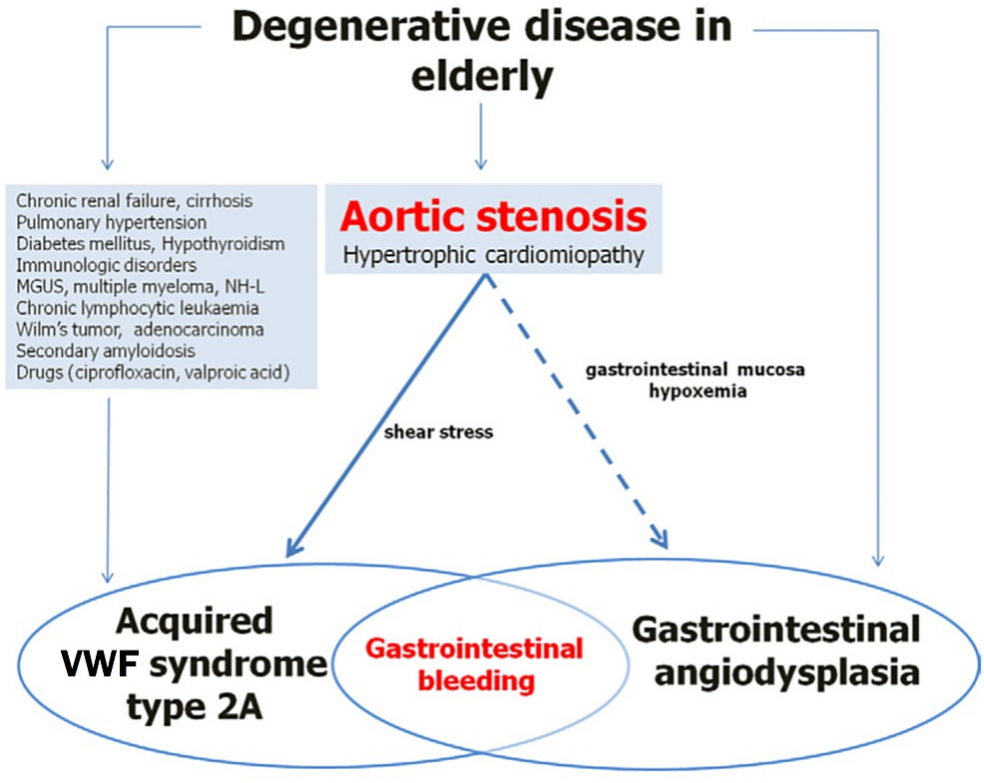
Pathogenesis of Heyde's syndrome. Aortic valve stenosis and other degenerative diseases in elderly patients can favor gastrointestinal bleeding through intricate mechanisms. First of all, aortic stenosis would give rise to GIB by reducing gastrointestinal perfusion and leading to hypoxemia-induced dilation of blood vessels (link between aortic stenosis and gastrointestinal angiodysplasia). Moreover, it was suggested that in patients with aortic stenosis, GIB would be favored by acquired type 2A Von Willebrand factor (VWF) syndrome (VWS 2A). Type 2A VWF syndrome is a subset of VWD; it is acquired secondary to aortic stenosis and is characterized by the lack of high molecular weight VWF multimers. Other degenerative disorders can predispose to VWS 2A, which is characterized by decreased level of high molecular weight VWF multimers impairing platelet adhesion to the subendothelium. In this setting, GIB is caused by the coexistence of the VWS 2A and gastrointestinal angiodysplasia. This image is reprinted with permission from Godino et al. [8]. GIB: gastrointestinal bleeding; VWF: Von Willebrand factor; VWS: Von Willebrand factor syndrome; MGUS: monoclonal gammopathy of undetermined significance; NH-L: non-Hodgkin’s lymphoma

To fully grasp the pathophysiology of Heyde's syndrome, one must be aware that it is a complex disease commonly seen in the elderly which involves an interplay between aortic stenosis, gastrointestinal angiodysplasia, and acquired Von Willebrand's disease. Figure 2 illustrates the pathogenesis of Heyde’s syndrome in which the downstream effects of aortic stenosis induce gastrointestinal angiodysplasia and acquired VWF syndrome type 2A [8].

Angiodysplasia: Angiodysplasia refers to an acquired vascular aberration in which the blood vessels of the GI tract form pathological tortuous arteriovenous (AV) malformations in the mucosa and submucosa [1,2,9]. It is the second most common cause of developing a lower GI bleed in patients over the age of 60 years [10]. The exact mechanism by which angiodysplasia develops is not completely understood; however, there are multiple proposed etiologies [11]. According to Boss and Rosenbaum, who examined postmortem bodies of patients with aortic stenosis, low-grade hypoxia was a possible mechanism for the intestinal mucosal vessel distension. The hypoxia triggered a reflex sympathetic response leading to chronic vasodilation, thus causing angiodysplasia to develop [12]. Low-grade hypoxia can also induce angiogenesis leading to neovascularization. Moreover, studies show that VWF plays an inhibitory role in the signaling of vascular endothelial growth factor (VEGF), which is an integral angiogenic growth factor. Loss of high molecular weight VWF multimers in Heyde's syndrome leads to increased VEGF activity and, as a result, enhanced vascular proliferation [6]. Newly formed blood vessels are fragile and may be more vulnerable to GI bleeding [10].

Acquired VWD: VWD can be classified according to three major types, type 1 (partial), type 2 (qualitative), or type 3 (total). Heyde's syndrome is characterized by a subset of type 2 VWD called type 2A, which exhibits a lack of high molecular weight (HMW) VWF multimers [13]. VWF is stored in Weibel-Palade bodies and the alpha granules of platelets after its synthesis in endothelial cells and megakaryocytes [10,13]. These granules contain large VWF multimers, which are then cleaved into low and high molecular weight forms. High molecular weight multimers play an active role in hemostasis [6,13]. Aortic stenosis causes increased shear stress on the VWF multimers, this is due to the increased speed at which blood traverses through the valve. This shear stress leads to elongation of the VWF multimers [6,10]. These multimers typically exist in a closed globular conformation; when elongation occurs, hidden cleavage sites are exposed, making them more susceptible to proteolytic cleavage via a disintegrin and metalloproteinase with a thrombospondin type 1 motif, member 13 (ADAMTS13) [6,14], and thus the remaining small VWF multimers are unable to induce adequate hemostasis making patients more susceptible to GI bleeding [10]. The incidence of acquired VWD is not at all uncommon; one study showed that 79% of patients with severe aortic valve stenosis had decreased levels of high molecular weight VWF multimers [6].

Aortic stenosis: Aortic stenosis is a relatively common condition, especially in the elderly, as it is seen in 1.3% of patients over the age of 65 years; for patients over the age of 85 years, this figure rises to 4% [4]. Furthermore, it represents the leading cause of valvular heart disease in the west [15]. The association between angiodysplasia and aortic stenosis is well documented; according to a study done between 1990 and 2000, out of 73 patients with confirmed angiodysplasia, 31.7% were shown to have aortic stenosis as compared to mitral stenosis at 14% [10]. The survival rate in asymptomatic patients with aortic stenosis is roughly identical to that of age- and sex-matched controls. However, the survival rates in symptomatic patients are far lower [16].

Aortic stenosis can be caused by various conditions like autoimmune disorders, bicuspid aortic valve, diabetes mellitus, and hypercholesterolemia, to name a few. These conditions can induce inflammatory stress on the aortic valve which leads to subsequent fibrotic changes that progress to valvular calcification [17]. Aortic stenosis can produce findings such as angina, left ventricular remolding, heart failure, and in the case of Heyde's syndrome, an increased risk of GI bleeding.

Diagnostic Investigations

When diagnosing Heyde's syndrome, relevant investigations should be done for the system affected.

Cardiovascular: For aortic stenosis, a transthoracic echocardiogram is the modality of choice to diagnose and assess the extent of the disease [12,18]. It can visualize valve leaflets, their motion, calcification, and left ventricular function [16].

Gastrointestinal: Depending on the situation, the presence or absence of, and the degree of bleeding, multiple methods can be used to diagnose angiodysplasia [11,18]. Upper and lower GI endoscopy and colonoscopy are the primary modalities used for the diagnosis of angiodysplasia located in the upper GI tract and colon; this is in part due to the technological advancements which have aided operators by allowing them to get more precise visualization of the gastrointestinal tract [11].

The ability to adequately visualize angiodysplasia (AD) in the small bowel can vary depending on factors such as operator skill, lack of adequate visibility, and misdiagnosis. Wireless capsule endoscopy is a valid alternative as it can provide the same or higher diagnostic acuity and yet is less invasive than enteroscopy and angiography [11].

Radiological investigations can be undertaken in patients who present with symptoms of an overt GI bleed in order to diagnose or confirm its location. These range from modalities such as CT and MR angiography to standard angiography and radionucleotide scanning. Standard angiography, as compared to the others, has the advantage of allowing the radiographer to pinpoint the location of the bleed in addition to performing a therapeutic embolization.

Diagnosis of Acquired VWD

VWD can be diagnosed via ristocetin cofactor activity or gel electrophoresis, which represents the gold standard for diagnosing acquired Von Willebrand's disease. These tests; however, are not routinely used due to cost, lack of accessibility, and time taken to yield results. A platelet function analyzer is a point of care test with high sensitivity when it comes to detecting VWD [6]. Furthermore, it can produce rapid results and can therefore be used as a screening test [6].

Surgical/interventional and medical management

Regarding the management of Heyde's syndrome, there are no established guidelines for treatment; the approach to management is based on managing GI bleeding and the treatment of aortic stenosis and VWD.

GI Bleeding

For GI bleeding, the approach may vary depending on whether the patient is actively bleeding or not. Asymptomatic patients in which incidental angiodysplasia signs are found have a low risk of developing a bleed in the future and, as a result, do not need to undergo treatment [1,11]. However, treatment can be considered in patients with a history of angiodysplasia who present with symptoms of occult GI bleeding [1,11]. In the case of active bleeding, the patient's hemodynamics must be maintained either via IV fluid or blood transfusions [10]. Angiography can then be used to localize the bleed, followed by embolization [1]. Another method that can be used is endoscopic laser photocoagulation; however, rebleeding can occur in roughly one-third of patients [12]. Furthermore, endoscopic management via argon plasma coagulation is another viable treatment modality.

Medical Management-VWD

In Heyde's syndrome patients, VWD is caused due to the shear stress exerted by the stenosed aortic valve and as a result, patients are predisposed towards developing recurrent GI bleeding. Managing VWD in the setting of Heyde's can be approached in one of two ways - by administration of medication that increases the amount of circulating VWF in the blood or by administration VWF containing products like factor concentrates and fresh frozen plasma [6]. Medical management in the form of desmopressin is a prime example of the former. Desmopressin increases levels of VWF by stimulating the release of VWF from endothelial cells [10]. One study done by Steinlechner et al. showed that preoperative desmopressin administration led to a 42% reduction in post-operative bleeding in patients with severe aortic valvular stenosis who were supposed to undergo surgical aortic valvular replacement [3]. Somatostatin is another viable form of medical management for Heyde's syndrome patients. It decreases portal venous pressure, which can subsequently reduce gastrointestinal blood loss [12].

Aortic Valvular Replacement

For definitive management, patients need to undergo aortic valvular replacement [9,10,17,18,19] as it can lead to the normalization of high molecular weight VWF multimer levels [6,19]. Valvular replacement can be done either via surgical replacement or via TAVR [9]. TAVR has steadily become a widely accepted treatment option for aortic stenosis. One nationwide study showed that patients undergoing TAVR had fewer incidences of perioperative MI, stroke, and blood transfusion compared to their surgical aortic valve replacement (SAVR) counterparts [6]. Additionally, a retrospective analysis conducted between 1971 and 2007 identified 57 patients with GI bleeding secondary to angiodysplasia, all of whom underwent aortic valve replacement. Out of these patients, 45 had no bleeding recurrence over the course of a 15-year follow-up (79%) [3]. This supports the notion that the hematological aberration observed in Heyde's syndrome patients is induced by aortic stenosis, and hence replacement can be curative. In spite of this, post-valve replacement paravalvular leakage has been shown to be the most substantial risk factor for the reoccurrence of GI bleeding as a result of residual shear stress preventing normalization of multimer levels [6,20].

Limitations

There are numerous limitations to this systematic review; first and foremost, most of the studies included in this paper had a limited number of patients due to the lack of widespread prevalence of Heyde's syndrome. Furthermore, this systematic review is limited to free articles published within the last 10 years; therefore, critical studies outside this timeframe that could have substantially contributed to this systematic review were not implemented. Lastly, regarding the management of Heyde's syndrome, this study mainly focused on interventional/procedural management as opposed to medical management due to limited information existing on the medical management of Heyde's syndrome.

## Conclusions

This study aimed to give a systematic overview to enlighten the reader about the pathophysiology, diagnostic and multimodal management strategies for Heyde's syndrome patients. Treatment strategies ranged from aortic valvular replacement, which was found to be the most definitive treatment modality in some studies, to GI endoscopic treatments such as argon plasma coagulation. Furthermore, various diagnostic modalities were highlighted, such as those for aortic stenosis, in which echocardiography is the gold standard. For Von Willebrand's disease, the platelet function analyzer is a valuable point of care diagnostic test. This paper is crucial as it draws attention to a condition that is undoubtedly underdiagnosed and, as a result, underreported in the literature. Hence it can serve to educate primary care providers and specialists alike on this disease. For future trials, we would recommend that clear-cut guidelines are established for the management of patients diagnosed with Heyde's syndrome as, so far, none exist, and as a result, patients are treated on a case-by-case basis. Furthermore, the cohort of patients in which researchers assessed the efficacy of valvular replacement in Heyde's syndrome patients was notably relatively small; hence we recommend that future studies include a larger group of patients.
